# Improving WHO’s understanding of WHO guideline uptake and use in Member States: a scoping review

**DOI:** 10.1186/s12961-022-00899-y

**Published:** 2022-09-07

**Authors:** Kiran Saluja, K. Srikanth Reddy, Qi Wang, Ying Zhu, Yanfei Li, Xiajing Chu, Rui Li, Liangying Hou, Tanya Horsley, Fred Carden, Kidist Bartolomeos, Janet Hatcher Roberts

**Affiliations:** 1 Bruyere Research Institute, Ottawa, Canada; 2 School of Epidemiology and Public Health, Faculty of Medicine, University of Ottawa, 600 Peter Morand Crescent, Ottawa, ON K1G 5Z3 Canada; 3 Department of Health Research Methods, Evidence and Impact, McMaster University, Hamilton, Canada; 4 Evidence Based Medicine Center, School of Basic Medical Sciences, Lanzhou University, Lanzhou, China; 5 Evidence Based Social Science Research Center, School of Public Health, Lanzhou University, Lanzhou, China; 6 Royal College of Physicians and Surgeons of Canada, Ottawa, Canada; 7 Using Evidence Inc., Ottawa, Canada; 8 Science Division, World Health Organization, Geneva, Switzerland; 9 WHO Collaborating Centre for Knowledge Translation and Health Impact Assessment in Health Equity, Bruyere Research Institute, University of Ottawa, Ottawa, Canada

**Keywords:** World Health Organization, Guidelines, Implementation, Evaluation, Uptake, LMICs

## Abstract

**Background:**

WHO publishes public health and clinical guidelines to guide Member States in achieving better health outcomes. Furthermore, WHO’s Thirteenth General Programme of Work for 2019–2023 prioritizes strengthening its normative functional role and uptake of normative and standard-setting products, including guidelines at the country level. Therefore, understanding WHO guideline uptake by the Member States, particularly the low- and middle-income countries (LMICs), is of utmost importance for the organization and scholarship.

**Methods:**

We conducted a scoping review using a comprehensive search strategy to include published literature in English between 2007 and 2020. The review was conducted between May and June 2021. We searched five electronic databases including CINAHL, the Cochrane Library, PubMed, Embase and Scopus. We also searched Google Scholar as a supplementary source. The review adhered to the PRISMA-ScR (PRISMA extension for scoping reviews) guidelines for reporting the searches, screening and identification of evaluation studies from the literature. A narrative synthesis of the evidence around key barriers and challenges for WHO guideline uptake in LMICs is thematically presented.

**Results:**

The scoping review included 48 studies, and the findings were categorized into four themes: (1) lack of national legislation, regulations and policy coherence, (2) inadequate experience, expertise and training of healthcare providers for guideline uptake, (3) funding limitations for guideline uptake and use, and (4) inadequate healthcare infrastructure for guideline compliance. These challenges were situated in the Member States’ health systems. The findings suggest that governance was often weak within the existing health systems amongst most of the LMICs studied, as was the guidance provided by WHO’s guidelines on governance requirements. This challenge was further exacerbated by a lack of accountability and transparency mechanisms for uptake and implementation of guidelines. In addition, the WHO guidelines themselves were either unclear and were technically challenging for some health conditions; however, WHO guidelines were primarily used as a reference by Member States when they developed their national guidelines.

**Conclusions:**

The challenges identified reflect the national health systems’ (in)ability to allocate, implement and monitor the guidelines. Historically this is beyond the remit of WHO, but Member States could benefit from WHO implementation guidance on requirements and needs for successful uptake and use of WHO guidelines.

**Supplementary Information:**

The online version contains supplementary material available at 10.1186/s12961-022-00899-y.

## Contributions to the literature

- Member States’ health systems determine WHO guideline uptake; weaker health systems continue to have low uptake and use of WHO guidelines.
- The challenges for WHO guideline uptake reflect the health systems’ (in)ability to allocate, implement and monitor adherence to the guidelines. Historically this is beyond the remit of WHO, but Member States could benefit from WHO implementation guidance on requirements and needs for successful deployment of normative and standard-setting products.
- Robust feedback mechanisms between WHO and Member States help to optimize WHO guideline uptake in Member States and contribute to the guideline development process.

## Background

WHO has a long tradition of supporting the Member States in developing national health policies, strategies and plans through country-level technical cooperation, facilitation of national policy dialogue and inter-country exchange, as well as through its normative work, including the provision of guidelines [1]. WHO defines a guideline as any document developed by WHO containing recommendations for clinical practice or public health policy. These guidelines outline recommendations for *end-users* regarding what can or should be done in specific situations to achieve the best health outcomes possible. Guidelines are the fundamental means by which the organization fulfils its technical leadership role in health [2].

Low- and middle-income countries (LMICs) often lack resources and/or skills to develop local guidelines and instead rely on guidelines developed by WHO and other international organizations [3]. Constraints in guideline development in LMICs include methodological problems and inadequate resources [4, 5]. Scholars have critically argued that the adoption of guidelines in LMICs, in merely attempting to emulate “*clinical guidelines developed in rich countries, risks placing unnecessary strains on their health services*” [6]. WHO plays a critical role in addressing the need for evidence-informed guidance for the Member States, particularly LMICs. For example, WHO guidelines provided a valuable reference for establishing new national regulatory requirements or updating existing ones and promoting convergence at the global level to enable regulatory cooperation for biotherapeutics among the Member States [7].

WHO’s Thirteenth General Programme of Work (GPW13) also prioritizes strengthening its normative functional role and uptake of normative and standard-setting products (NSPs) inclusive of guidelines at the country level [8]. As such, WHO policy-makers and guideline developers seek to understand the extent of uptake and how the guidelines are integrated into the policy and practice in LMICs, where the maximum use of WHO guidelines is expected. However, the literature reveals limited evidence evaluating the uptake, use and impact of WHO guidelines [3, 9–11]. WHO has also echoed a limited understanding of the uptake and use of NSPs by the Member States, and aimed to understand the barriers to uptake and use and determinants of success of WHO’s NSPs at the policy and practice levels in LMICs [12].

Since 2007, WHO’s Guidelines Review Committee (WHO GRC) has engaged in defining the standards and methods for all guidelines that are funded, developed and issued by WHO, and follows rigorous methods of development to ensure its recommendations are evidence-based [13]. To optimize uptake and use of WHO’s GRC-approved guidelines, WHO commissioned a review of the literature to contribute to what is known about the uptake and use of WHO guidelines in LMICs. The review findings were intended to inform WHO about existing evidence around barriers to guideline uptake and to support WHO’s Department of Quality Assurance, Norms and Standards activities (QNS), particularly in strengthening the framework for monitoring, evaluation and learning on the uptake and use of WHO norms and standards in LMICs. Accordingly, we conducted a scoping review to summarize evidence on the barriers to uptake of WHO’s clinical and public health guidelines at the policy and practice levels amongst LMICs, thereby contributing to WHO’s understanding of its guideline uptake.

## Methods

A scoping review is defined as a type of research synthesis that aims to “*map the literature on a particular topic or research area and provide an opportunity to identify key concepts; gaps in the research; and types and sources of evidence to inform practice, policymaking, and research*” [14, 15]. We conducted a scoping review between May and June 2021, to identify and synthesize the evidence around barriers to uptake of WHO GRC-approved guidelines in LMICs. Due to significant heterogeneity in the aspects of guideline topics, implementation interventions, study design and outcomes across the included studies, the project advisory committee determined that using a meta-analysis may not have been worthwhile for pooling the quantitative data. Further, the use of a narrative synthesis was recommended as more appropriate for presenting the themes and subthemes in the scoping review.

### Search strategy

The search strategy aimed to identify published articles that evaluated WHO guideline uptake in LMICs. We searched five electronic databases including CINAHL, the Cochrane Library, PubMed, Embase and Scopus. We also searched Google Scholar as a supplementary source. The search was limited to studies published between January 2007 (inception of WHO GRC) and December 2020. We also reached out to the WHO departments and experts in implementation science to retrieve relevant published or grey literature. The search strategy for the scoping review, including a detailed list of search terms, was developed and finalized in consultation with the project advisory committee members and the WHO team. The detailed search strategies for each database are included in Additional file 1.

### Study selection criteria

Studies were included if they evaluated country-specific adaptation/adoption/contextualization, implementation and uptake/use of WHO GRC-approved clinical practice and public health guidelines within LMICs. Because of the limited time frame for this review, the selection of records was limited to studies reporting on guidelines for specific health conditions including nutrition; maternal, newborn and child health (MNCH); communicable diseases; noncommunicable diseases (NCDs) and neglected tropical diseases. These health conditions were selected in consultation with the project advisory committee based on the disease burden in LMICs. Further, as health conditions were broad, the project advisory committee advised the project team to limit their review to specific diseases. Accordingly, for communicable diseases, we have included only the big three infectious diseases—HIV, tuberculosis (TB) and malaria—and for neglected tropical diseases, which comprise a diverse group of 20 tropical infections, we have included only filariasis and schistosomiasis. While there was no restriction on study design for the inclusion of studies in the review, we excluded records that were not published in the English language and those that were purely descriptive, as well as those that did not formally evaluate WHO guidelines or their components. For our selection criteria, “purely descriptive” studies are studies that describe or present the implementation process of and/or experience with guidelines without any assessments or evaluations through quantitative, qualitative or mixed approaches.

### WHO guideline and descriptor terms

Scholars have noted that the titles of WHO guidelines often include a variety of descriptor terms other than “guidelines” itself [16], rendering it difficult to identify WHO guidelines. A recent study on WHO guidelines reported considerable variation in descriptor terms used for the WHO documents, including guideline(s), recommendation(s), guidance, policy statements and a variety of other terms (manual, rapid advice, handbook, statement, guide, toolkit, technical paper) [16]. In order to develop our screening criteria and to determine whether the descriptor reported in the articles qualified as a WHO guideline, we obtained from the WHO QNS team a comprehensive list of GRC-approved WHO guidelines recorded in the WHO Institutional Repository for Information Sharing (IRIS) database to identify all the terms used to define WHO guidelines. The data retrieved from the IRIS database included 439 WHO GRC-approved guidelines published between 2007 and 2020, 62% of which (*n* = 273) were in English. These guidelines have used varied descriptor terms such as guidelines (*n* = 151), recommendation/s (*n* = 5), policy guidance (*n* = 8), policy statement (*n* = 6), guidance (*n* = 5) and others (*n* = 98). Our review team screened and categorized these guidelines as per the preselected health conditions considered for this review (Table 1). The final list guided our screening criteria.

**Table 1:** The list of health conditions for the 273 English-language guidelines

Health condition/theme	No.	Health condition/theme	No.
Air pollution	4	Mental health	10
Anthrax	1	MNCH-A	55
Blood donation	4	NCD	8
Chlamydia	1	NCD/MNCH-A	1
Dengue	1	NCD/SRH	1
Disability	1	*Neisseria gonorrhoeae*	1
Drinking water	1	Nutrition	14
Drug	4	Nutrition/MNCH-A	7
Drug/substance use	1	Nutrition/SRH	10
Ebola	2	Rehabilitation	2
Filariasis	1	Rehabilitation/health services	1
*Trypanosoma brucei gambiense* human African trypanosomiasis	1	Respiratory	1
Health services	22	Smoking	1
Helminthiasis	2	SRH	14
Hepatitis	8	SRH/health services	3
Herpes Genitalis	1	SRH/HIV	1
HIV	35	SRH/MNCH-A	1
Influenza	2	Substance use	2
Lung disease	1	Syphilis	1
Malaria	2	TB	38
Measles	2	Telemedicine	1
Meningitis	1	Zika	2

*MNCH* maternal, newborn and child health, *MNCH-A* MNCH–antenatal care, *NCD* noncommunicable disease, *SRH* sexual and reproductive health

### Eligibility assessment and data extraction

All records identified through searches were uploaded to a proprietary review management software programme (Covidence), and duplicate references were identified and subsequently removed. Four authors (KS, KSR, QW, YZ) piloted the study selection process on a sample of records (*n* = 20) based on the predefined inclusion/exclusion criteria, and inter-rater agreement was assessed. When sufficient agreement was reached, the titles and abstracts for all the records were independently screened by two authors to identify eligible articles. Disagreements at this stage were resolved through discussion among the four reviewers. Full-text records were retrieved and reviewed for inclusion by a single author and verified by a second author. The review team followed the Preferred Reporting Items for Systematic Reviews and Meta-Analyses extension for scoping reviews (PRISMA-ScR) for reporting decisions for inclusion/exclusion and reporting of review items [17–20]. Once inclusion was established, data were extracted for each study using a pre-piloted data extraction form. The data extraction items included the author(s), publication year, study location (name of LMICs), the WHO guideline(s) of interest, the condition(s)/topics, aims/objectives of the study, study design, barriers, facilitators/enablers, implementation interventions/suggestions/recommendations and implementation frameworks. Four authors (KS, KSR, QW, YZ) separately extracted the above items from the included studies, and another two senior authors (KS, KSR) further randomly checked the extracted data for imprecision. Based on the finalized data extraction from the included studies, focusing on our research questions, we categorized the themes and related subthemes and organized their relationships through multiple group discussions. We stopped adding new themes and subthemes upon data saturation [21, 22].

## Results

**Fig. 1:**
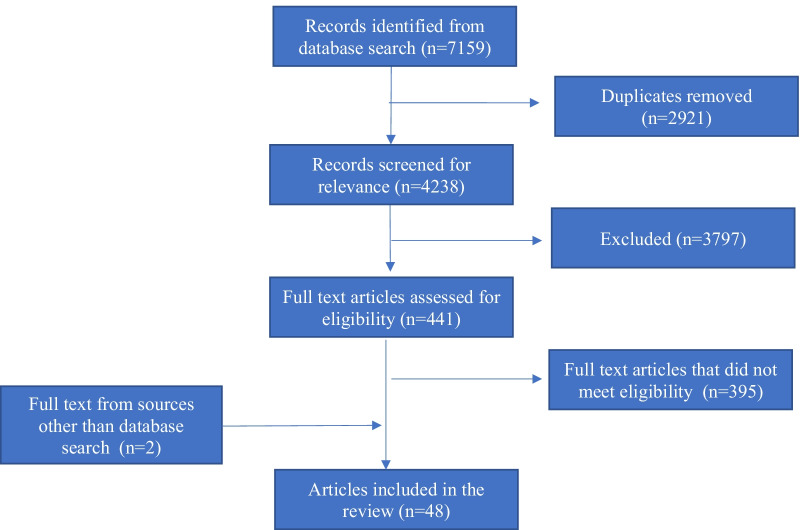
Scoping review study flow diagram

Forty-eight studies were identified as eligible for inclusion following full-text assessment (*n* = 48/7159) (Fig. 1). Two records were further identified as eligible for inclusion following reference list screening of the included records.

The detailed characteristics of the included studies and findings are presented in Table 2. The review findings reveal key barriers for uptake and use of WHO GRC guidelines and are thematically categorized as (1) lack of national legislation, regulations and policy coherence, (2) inadequate experience, expertise and training of healthcare providers for guideline uptake, (3) funding limitations for guideline uptake and use, and (4) inadequate healthcare infrastructure for guideline compliance. For each thematic area, we present key examples of reported barriers to WHO GRC guideline uptake.

**Table 2:** Studies on WHO guideline uptake and use included in the review

Author	Year	Country	Study design	Health condition	Guideline	Barriers
Chanda et al.	2015	Malawi	Case study	Malaria	WHO-recommended indoor residual spraying for malaria transmission control and elimination	Limited funding, cost of alternative insecticides and technical resource challenges
Chinkonde et al.	2010	Malawi	Qualitative study	HIV and infant feeding	UNAIDS, WHO, UNFPA and UNICEF guidelines for HIV and infant feeding	Lack of consensus and general confusion regarding guidelines at all levels, need for resources, lack of up-to-date information, lack of contextualized and easy-to-follow guidelines
Church et al.	2015	Kenya, Malawi, South Africa, Uganda, the United Republic of Tanzania and Zimbabwe	Mixed methods study	HIV	HIV testing and treatment guidelines	Lack of WHO explicit guidance around pre-ART CD4 monitoring intervals, rapid initiation of ART, task-shifting for ART initiation, drug resupply intervals, pill count recommendations, drug collection by designees, referral to peer support and home-based care
Doherty et al.	2007	South Africa	Prospective cohort study	Infant feeding practices/HIV mothers	WHO/UNICEF guidelines on infant feeding for HIV-positive women. The guidelines recommend that HIV-positive women avoid all breastfeeding only if replacement feeding is acceptable, feasible, affordable, sustainable and safe	Within operational settings, the WHO/UNICEF guidelines were not being implemented effectively, leading to inappropriate infant feeding choices and consequent lower infant HIV-free survival
Finocchario-Kessler et al.	2016	Kenya	Retrospective cohort study	HIV	Antiretroviral drugs for treating pregnant women and preventing HIV infection in infants: recommendations for a public health approach—2010 version	Inadequate and inconsistent training, less efficacious regimens, weak systems for patient follow-up and retention
Govere and Chimbari	2020	Sub-Saharan Africa	Scoping review	HIV	WHO’s CD4-threshold ART initiation recommendations	Economic constraints, drug stock-outs, delays in obtaining baseline blood test results and staff shortages
Hodges-Mameletzis et al.	2018	LMICs	Descriptive policy review	HIV	Pre-exposure prophylaxis (PrEP) containing tenofovir disoproxil fumarate (TDF)	Underlying cost of PrEP services, cost considerations include commodities, from the drug itself to the additional testing required to ensure PrEP is offered effectively and safely
Jones-López et al.	2011	Uganda	Prospective cohort study	TB	Standard WHO-recommended retreatment regimen (category II) for TB	Lack of access to rapid diagnostics for TB drug resistance, second-line TB treatment, ART and limited guidance among the policy-makers and healthcare providers on using the tools available
Lecher et al.	2015	Sub-Saharan African countries (Côte d’Ivoire, Kenya, Malawi, Namibia, South Africa, Tanzania, Uganda)	No design specified	HIV	Consolidated guidelines on the use of antiretroviral drugs for treating and preventing HIV infection: recommendations for a public health approach	Lack of trained laboratory personnel, no operating budget, difficulty transporting samples, delays in commodity procurement and distribution, inadequate laboratory information systems, insufficient trained human resources, equipment breakdown, delay in equipment repair, inadequate laboratory and storage space, insufficient viral load testing results management
Nadjm et al.	2010	Tanzania	Observational study	Malaria	WHO manual *Management of the child with severe infection or severe malnutrition: guidelines for care at first-referral level*. This is the standard WHO guide for paediatric inpatient care and has been adopted as policy by the ministries of health of many resource-poor countries	In an area exposed to high transmission of malaria, current WHO guidelines failed to identify almost a third of children with invasive bacterial disease, and more than half of the organisms isolated were not susceptible to currently recommended antimicrobials. Improved diagnosis and treatment of invasive bacterial disease is needed to reduce childhood mortality
Nasser et al.	2015	20 LMICs in Africa and South-East Asia	Logistic regression analysis	HIV and TB guidelines	WHO HIV and TB guidelines	
Ngoma et al.	2015	Zambia	Prospective observational cohort study	HIV	WHO guidelines recommend maternal combination antiretroviral therapy (cART) during pregnancy, throughout breastfeeding for 1 year and then cessation of breastfeeding (COB)	Maternal cART may limit mother-to-child transmission of HIV to the UNAIDS target of <5% for eradication of paediatric HIV within the context of a clinical study, but poor adherence to cART and follow-up can limit the benefit
Stanecki et al.	2010	No country specified	No design specified	HIV	WHO guidelines on ART for HIV infection in adults and adolescents	
Stover et al.	2014	24 LMICs	Modelling	HIV	WHO ART treatment guidelines	Mobilizing additional resources; expanding facilities, personnel and drug supply chains; and identifying HIV-infected people at higher CD4þ T-cell counts and those in serodiscordant partnerships; the large number of patients
Tlhajoane et al.	2018	Zimbabwe	Longitudinal study	HIV	WHO recommendations on HIV testing services, prevention of mother-to-child transmission (PMTCT) of HIV, and provision of ART	Limited availability of different regimen choices posed challenges in the provision of ART stock-outs; laboratory monitoring remained confined to larger hospitals
Tudor Car et al.	2013	LMICs	Systematic review	HIV	1. WHO (2010) PMTCT strategic vision 2010–2015: preventing mother-to-child transmission of HIV to reach the UNGASS and Millennium Development Goals 2. Technical consultation on the integration of HIV interventions into maternal, newborn and child health services 3. WHO (2005) Glion consultation on strengthening the linkages between reproductive health and HIV/AIDS: family planning and HIV/AIDS in women and children	Late admission, unknown HIV status, fear of stigma, the policy context
Van Deun et al.	2020	LMICs	Review	TB	WHO treatment guidelines for multidrug- and rifampicin-resistant TB. 2018 update	The drug susceptibility testing capacity, long regimen
Downs et al.	2015	India	Mixed-methods	NCDs	WHO recommends virtually eliminating trans fat from the global food supply	
Dzudie et al.	2020	Cameroon	Survey	NCDs/CVD	WHO’s 25 × 25 goal is aimed at achieving a 25% reduction in the number of premature deaths (occurring before 70 years of age) due to NCDs by 2025	Availability of essential medicines for CVD was 33%, much lower than the 80% recommended by the WHO Global Action Plan for Prevention of NCDs
Kaltenbrun et al.	2020	South Africa	Qualitative	NCDs and nutrition	WHO recommends that countries adopt a fiscal policy to reduce the consumption of sugar-sweetened beverages	Political will, limited delivery capacity, legislation restrictions and competing government priorities
Pati et al.	2020	India	Narrative review	NCDs	NCD Global Monitoring Framework	Challenges in the identification of eligible beneficiaries, shortage and poor capacity of frontline health workers, poor functioning of community groups and poor community knowledge on NCD risk factors were key gaps at the community level. Challenges at the facility level such as poor facility infrastructure, lack of provider knowledge on standards of NCD care and subpar quality of care led to poor management of NCDs. At the health system level, organization of care, programme management and monitoring systems were not geared up to address NCDs. Multisectoral collaboration and coordination were proposed at the policy level to tackle NCDs; however, gaps remained in implementation of such policies
Abebe et al.	2019	Ethiopia	Cross-sectional study	MNCH/neonatal	Integrated Management of Neonatal Childhood Illness (IMNCI) strategy	Shortage of essential drugs and supplies, inadequate trained staff, time-consuming nature of the protocol, lack of supervision, lack of knowledge about the strategy and lack of good attitude among healthcare workers/professionals towards the IMNCI strategy
Ansah Manu et al.	2014	Ghana	Cluster-randomized trial	MNCH	Home visits for the newborn child: a strategy to improve survival: WHO/UNICEF joint statement	Poor facility, poor health worker attitudes
Braddick et al.	2016	Uganda	Mixed methods	MNCH	WHO PPH guideline: adherence to AMTSL guidelines according to WHO PPH recommendations	Healthcare system issues; current knowledge, awareness, and use of clinical guidelines; and healthcare practitioner attitudes towards updating their clinical practice
Chang et al.	2020	Bangladesh	Mixed methods	MNCH	WHO’s 2016 standards for improving quality of maternal and newborn care in health facilities	The volume of existing indicators
Chu et al.	2012	LMICs	Review	MNCH	Misoprostol use to prevent and treat PPH	Research evidence does not support misoprostol use in home and community settings in LMICs for PPH prevention. WHO should rethink its recent decision to include misoprostol on the essential medicines list
Colvin et al.	2013	Lower- and middle-income countries	Systematic review	MNCH	WHO recommendations: optimizing health worker roles to improve access to key maternal and newborn health interventions through task-shifting guidelines	Lack of legal protection and liabilities and the regulatory framework for task-shifting
Doku and Neupane	2017	57 LMICs	Cross-sectional Demographic and Health Survey	MNCH–ANC	WHO recommendations for ANC (first visit within the first trimester and at least four visits during pregnancy)	
Downe et al.	2019	South Africa, Indonesia, United Kingdom, Papua New Guinea, Australia, Peru, Uganda, Ghana, United States, Brazil, Ethiopia, Mozambique, Nigeria, Bangladesh, Argentina, Kenya, Iran, Viet Nam, India, Tanzania, Canada, Ireland, Lebanon, Pakistan, Australia, New Zealand, Sweden, Colombia, Romania, Lao People’s Democratic Republic, Zimbabwe, Cambodia, Peru, Georgia, South Sudan, Afghanistan, Iraq, Nepal, Gambia, Swaziland, France, Burkina Faso	Review	MNCH	WHO recommendations on ANC for a positive pregnancy experience	Inconvenience of clinic attendance, lack of accessibility and availability of local transport, indirect costs, potential loss of income, lack of privacy, lack of medicine and equipment, medical jargon
Khosla et al.	2017	Global	Review	MNCH	WHO developed the 2016 guidelines on the management of health complications from FGM	Lack of national legislation
Kraft et al.	2018	Ethiopia and Senegal	Qualitative study	MNCH/family planning	WHO’s evidence-based family planning guidance and tools (i.e. materials) that support the provision of quality family planning services	Resource constraints
Kumar et al.	2016	India	Quasi-experimental observational study	MNCH	WHO Safe Childbirth Checklist (SCC): the SCC targets high-impact best practices around four pause points that occur in almost every delivery: admission, pushing, just after delivery and pre-discharge	High-quality care provision at institutions is still a challenge
Mchenga et al.	2019	Malawi	Retrospective study	MNCH	2001 Focused Antenatal Care (FANC) programme	Unsupportive spouse, time lag
Nsabagasani et al.	2015	Uganda	Qualitative study	MNCH	WHO recommends the inclusion of child-appropriate dosage formulations in the essential medicines lists of member countries	Lack of resources
Ritchie et al.	2016	LMICs	Comparative study	MNCH	WHO guidelines on maternal, reproductive and women’s health	Lack of material and human resources, problems with communication and information sharing, policy issues, inadequate training, inadequate knowledge and skills, lack of access to evidence, lack of awareness of evidence, providers’ attitudes and beliefs, lack of financial resources, patients’ knowledge and beliefs, lack of communication, resultant lack of trust between providers and policy-makers, lack of accountability
Roberts et al.	2017	Malawi	Qualitative study	MNCH-A	2011 WHO statement on antenatal care	Beliefs, attitudes, control beliefs, and significant others; cultural beliefs adhered to by the mothers and providers, too many required antenatal care visits, travel distance
Samnani et al.	2017	developing countries	Systematic review	MNCH	Inclusion of misoprostol in its essential medicines list model in March 2011	Inconsistency in supplies and distribution; inadequate staffing; lack of knowledge of providers and end users, absence of the registration of drug, and fear and apprehension related to its use at the provider and policy levels. Leadership, governance and policy-related issues are substantial barriers to successful implementation of misoprostol in developing countries; fear and confusion among implementers, policy-makers and government officials; lack of awareness about existing policy; lack of integration of misoprostol in basic health service package
Shilton et al.	2019	Ethiopia	Mixed methods	MNCH	WHO guidelines on preventing early pregnancy and poor reproductive health outcomes among adolescents in developing countries	Knowledge, national agenda, laws, resources, culture, cooperation
Straus et al.	2013	Kosovo	Mixed methods	MNCH	WHO PPH guidelines	Lack of communication between clinicians and ministry representatives, substantial mistrust between clinicians and policy-makers, lack of communication across clinical groups that provide obstetric care and a lack of integration across the entire healthcare system, including rural and urban centres, inability to monitor quality of care, inability to consistently access required medications and to smoothly transfer patients from rural to urban centres
Vogel et al.	2016	Four LMICs—Myanmar, Uganda, Tanzania and Ethiopia	Mixed methods	MNCH	WHO maternal and perinatal health guidelines	Health system-level factors, including health workforce shortages and need for strengthened drug and equipment procurement, distribution and management systems, were consistently highlighted as limiting the capacity of providers to deliver high-quality care. Evidence-based health policies to support implementation and to improve the knowledge and skills of healthcare providers were also identified
Xue et al.	2020	LMICs	No design specified	MNCH/cervical	WHO calls for global action towards the elimination of cervical cancer; one of the main strategies is to screen 70% of women between the ages of 35 and 45 years and 90% of women managed appropriately by 2030, in order to achieve reduction to less than four new cases per 100,000 women	Shortage of experienced colposcopists, consummate colposcopy training courses, and uniform diagnostic standard and strict quality control
Zhang et al.	2017	74 Countdown countries	Modelling	MNCH	1. Guidelines for the management of common illnesses with limited resources 2. Community health worker manual, Facilitator notes 3. Guidelines for the management of common childhood illnesses, 2nd edition	NR
Ziegler et al.	2020	Democratic Republic of the Congo	Demographic and Health Surveys	MNCH	WHO recommendations on antenatal care for a positive pregnancy experience	Conflicts
Hossain et al.	2017	LMICs	Systematic review	Nutrition—severe acute malnutrition (SAM) in children	WHO’s facility-based guideline for the reduction of under-five SAM child mortality	High rates of poverty, malnutrition, severe comorbid conditions, lack of resources and differences in treatment practices
Mejia et al.	2019	The Americas: Chile, Costa Rica and Guatemala; Africa: Malawi, Uganda and Zambia; South Asia: Bangladesh; and the Western Pacific Region: China and the Philippines	Review	Nutrition	WHO recommends public health interventions to provide vitamins and minerals	Lack of regulatory frameworks, lack of safety measures

*AMTSL* active management of the third stage of labour, *ANC* antenatal care, *ART* antiretroviral therapy, *CVD* cardiovascular disease, *FGM* female genital mutilation, *MNCH* maternal, newborn and child health, *MNCH-A* MNCH–antenatal, *NCD* noncommunicable disease, *PPH* postpartum haemorrhage, *TB* tuberculosis, *UNAIDS* Joint United Nations Programme on HIV/AIDS, *UNGASS* United Nations General Assembly Special Session *UNICEF* United Nations Children’s Fund, *UNFPA* United Nations Population Fund

### Lack of national legislation, regulation and policy coherence

Public health legislation aims to promote or protect public health [23]. Our findings suggest that national public health legislation and regulations are pivotal to WHO guideline uptake and use in LMICs and a lack therein limits uptake. For example, in the context of communicable diseases, integrated vector management (IVM) is a vital component for controlling neglected tropical diseases and vector-borne diseases. In 2008, WHO issued a position statement supporting IVM consistent with the global strategic framework for IVM. However, one of the key reasons cited for the slow uptake of IVM was “*the lack of legislative activities*”, as implementation strategies for the IVM framework extend beyond the health sector. Therefore, intersectoral collaboration and establishment of regulatory and legislative control for public health and pesticide management, among others, were found critical for effective IVM programme implementation in malaria-endemic countries [24].

For NCDs, in response to the escalating burden of NCDs worldwide, the World Health Assembly (WHA) endorsed the WHO Global NCD Action Plan 2013–2020, which provides several evidence-based policy recommendations as “best buys” for NCD prevention and control. The Member States have adopted the NCD action plan; however, most best buy interventions were underutilized globally [25]. For example, the implementation of the WHO policy recommendations remained low in LMICs of Africa due to “*a lack of legislation and regulations for NCD control*” [26]. Scholars have noted that legislation and regulatory frameworks are critical for NCD prevention [27, 28]. The taxation on sugar-sweetened beverages with nutrition-sensitive agricultural policies can potentially improve overall health and nutrition in Africa [27]. However, lack of political will, legislative restrictions and competing government priorities were identified as major barriers to policy coherence in Africa and elsewhere [27, 29].

Another recent review noted that although multisectoral collaboration and coordination were proposed at the policy level to tackle NCDs in India, gaps remained in the implementation of such policies. The implementation gaps were reported at two levels: (1) at the intervention level, which included promoting physical activity in schools and society and restricting marketing of and access to food products high in salt, sugar or unhealthy fats, and (2) at the legislative level, which included clean indoor air legislation, tobacco advertising ban and raising the tax on tobacco products [28]. Similarly, another review found that “regulatory frameworks” were essential for public health interventions targeting nutrition [30]. The main types of regulatory frameworks include food safety and food fortification regulatory frameworks, among others, established by various international and national authorities. For example, the European Food Safety Authority for member countries of the European Union, the Food and Drug Administration in the United States, and the Health Products and Food Branch of Health Canada set the framework for the fortification of foods, along with the Regional Commission on Micronutrients and Fortified Foods in Central America and the National Commission on Micronutrients in Costa Rica, among others.

For MNCH, WHO recommends improving access to key maternal and newborn health interventions through task-shifting guidelines [31]. For the effective uptake and use of these recommendations, the legal protections and regulatory framework were found essential [32]. WHO guidelines also recommend preventing early pregnancy and poor reproductive health outcomes among adolescents in LMICs. However, guideline uptake in Ethiopia, for example, was constrained by “*lack of supporting laws and legislation along with other barriers*” [33]. Studies have reported that government policies prohibit the implementation of recommendations for postpartum haemorrhage (PPH) guidelines. For example, the administration of misoprostol by community healthcare workers was not supported by the policy in Uganda, despite studies conducted in Uganda demonstrating the safety and effectiveness of this approach under the supervision of midwives [34]. A similar lack of legal frameworks was found for the uptake of female genital mutilation (FGM) guidelines in most countries wherein FGM is practised [35].

### Inadequate experience, expertise, training and attitudes of healthcare providers

Healthcare providers play an invaluable role in healthcare delivery. Hence, the capacity-building of healthcare providers is essential in a health system. Advancement of knowledge and skills among practitioners is an important aspect of capacity-building [36]. WHO defines capacity-building as “*the development of knowledge, skills, commitment, structures, systems and leadership to enable effective health promotion…[with] actions to improve health at three levels: the advancement of knowledge and skills among practitioners; the expansion of support and infrastructure for health promotion in organizations; and, the development of cohesiveness and partnerships for health in communities*” [37].

For communicable diseases, studies found that the strength of recommendation and evidence quality determined national policy adoption of WHO HIV guidelines in LMICs in South-East Asia and Africa [38]. In contrast, a comparative analysis of HIV testing and treatment services in six sub-Saharan African countries argued that WHO did not provide explicit guidance on HIV testing and treatment services. As a result, countries had to move beyond WHO standards to formulate national HIV treatment policies. While there was insufficient guidance from WHO, the countries stipulated the need for periodic refresher training for healthcare providers on HIV prevention and treatment [39]. Other studies also reported that despite concerted efforts to provide treatment consistent with WHO guidelines, lack of health information and data integration have constrained the uptake and use of WHO’s guidelines for the prevention of mother-to-child transmission (PMTCT) of HIV in most LMICs [40, 41]. WHO and UNICEF [United Nations Children’s Fund] recommend that HIV-positive women avoid all breastfeeding only if replacement feeding is acceptable, feasible, affordable, sustainable and safe. However, the recommendations were not implemented effectively within operational settings in many African countries due to the lack of standardized health messaging for mothers to adopt the best practices, leading to inappropriate infant feeding choices and consequent lower infant HIV-free survival [42–44].

For MNCH, in 2018, WHO called for global action towards the elimination of cervical cancer, with a key strategy, among others, to screen 70% of women between the ages of 35 and 45 years. A study suggests well-organized screening programmes in high-income countries, but due to the lack of extensively experienced clinicians, LMICs did not achieve similar progress [45]. In 2011, WHO recommended misoprostol use to manage and prevent PPH in settings where oxytocin is not available and included misoprostol in its essential medicines list (EML) model. However, fear and confusion among policy-makers, programme managers and healthcare providers, lack of awareness about existing policy, and lack of integration of misoprostol in basic health service packages have been cited as substantial barriers to successful implementation of misoprostol administration in developing countries [46]. A review also highlighted that the research evidence does not support misoprostol use in home and community settings in LMICs for PPH prevention and indicated that WHO should rethink its decision to include misoprostol on the EML [47]. Nevertheless, the review suggested that government and policy-makers should focus on strengthening the health system and training birth attendants to prevent PPH in LMICs.

Though providers perceive PPH guidelines as useful, lack of guideline awareness, poor access to guidelines, prioritizing experience over evidence and incorrect clinical practice were key barriers to PPH guideline implementation in Kosovo [48]. In another example, descriptions of the guidelines were superficial and there were discrepancies as to which ones were used in clinical practice; limited access to guidelines (insufficient copies) at healthcare facilities, adherence to midwifery school-based knowledge rather than guidelines as best practices, and lack of knowledge about the rationale for using the guidelines (e.g. use of oxytocin) were cited [49]. In addition, lack of up-to-date guidance on recommended practices was highlighted as a challenge, particularly around the use of misoprostol for prevention of PPH in health facility settings in Uganda [34].

WHO recommends antenatal care (ANC) for a positive pregnancy experience for women, regardless of the income status of the country. However, lack of access to external training programmes was reported as a key barrier to compliance with ANC and PPH guidelines in LMICs [49–51]. In 2009, WHO and UNICEF issued a joint statement recommending home visits by community-based agents as a strategy to improve newborn survival. Evaluation studies conducted in LMICs cited poor health worker attitudes as barriers to uptake of the strategy [52] and optimal use of guidelines at the facility level [53].

### Funding limitations for guideline uptake and implementation

Adequate funding is essential for fulfilling the “*ten essential public health operations*” emphasized in WHO’s essential public health services framework [54]. However, LMICs continue to have limited public health funding and spending and rely on bilateral and multilateral assistance and other donor support. Our review suggests that the uptake and use of WHO guidelines in LMICs is significantly constrained by limited domestic public health funding and investments.

For communicable diseases, indoor residual spraying (IRS) is a proven effective malaria vector intervention if correctly implemented using WHO-recommended insecticides. Implementation of IRS programmes in malaria-endemic countries has often been constrained by funding limitations. For example, IRS programme implementation in Malawi was found to be uncertain due to limited funding, cost of alternative insecticides and technical resource challenges experienced in the country [55].

The 2013 WHO guidelines for antiretroviral therapy (ART) recommend expanding eligibility to include several new groups of people living with HIV, notably all HIV-infected adults with CD4þ T-cell counts between 350 and 500 cells/ml, all pregnant women and serodiscordant couples regardless of CD4þ T-cell count, and all HIV-positive children up to the age of 5 years. These guidelines were expected to double the number of people living with HIV/AIDS (PLHIV) on treatment, but several challenges limited its uptake in many countries [34, 53, 56]. The most common barriers to the timely implementation of new ART initiation guidelines were economic constraints for the procurement of drugs [57].

In 2015, WHO provided guidelines recommending that any person at substantial risk of HIV be offered oral pre-exposure prophylaxis (PrEP) containing tenofovir disoproxil fumarate (TDF) as an additional prevention choice. Further, in 2017, PrEP medicines were listed in WHO’s EML, including TDF/emtricitabine (FTC) and TDF in combination with lamivudine (3TC). By the end of 2018, at least 40 countries (20.6%) were anticipated to have adopted WHO’s oral PrEP recommendation. However, policy uptake and programmatic coverage of PrEP services were constrained by the underlying cost of PrEP services in LMICs [58]. Since 2015, WHO has also recommended a commercially available lateral-flow urine lipoarabinomannan (LAM) test (Alere-LAM) to assist in diagnosing TB in severely ill people living with HIV; however, the most commonly cited constraint to adoption and implementation of LAM was budget limitations [59].

For NCDs, WHO recommends virtually eliminating trans fat from the global food supply. LMICs such as India face several challenges, requiring a multisectoral food chain approach to remove trans fats from the food supply. Empirical evidence suggests that economic incentives for manufacturing foods using healthier oils are imperative in India and elsewhere [60]. In 2012, WHO set the 25 × 25 goal to achieve a 25% reduction in the number of premature deaths (occurring before 70 years of age) due to NCD by 2025. A global action plan followed this with a target of 80% availability and affordability of essential medicines for treatment and secondary prevention of cardiovascular disease (CVD) and other NCDs, and at least 50% of eligible people to receive drug therapy and counselling (including glycaemic control) to prevent heart attacks and strokes. A study reported a mean availability of essential medicines for CVD of 33%, much lower than the recommendation, and the available medicines were largely unaffordable, pointing towards the need for substantial investments in the LMICs [61].

For MNCH, in 2007, the 60th WHA passed a resolution entitled “Better medicines for children”. Subsequently, WHO recommended the inclusion of child-appropriate dosage formulations in the EMLs of Member States. However, LMICs have either delayed or not included these recommendations in their national EML. The key barriers included a lack of resources that hindered the formal transfer of the policy from the global to the local level [62]. A qualitative study conducted in Ethiopia and Senegal also found that while WHO’s evidence-based family planning guidance and tools were trustworthy, compliance was constrained by limited resources [63].

### Inadequate healthcare infrastructure for guideline compliance

Public health infrastructure provides the necessary foundation for undertaking the basic responsibilities of public health, which have been defined as the 10 essential public health operations [54]. Every public health programme requires health professionals who are competent in cross-cutting and technical skills, up-to-date information systems, and public health organizations with the capacity to assess and respond to community health needs. Public health infrastructure has been referred to as “the nerve center of the public health system” [64]. However, in most LMICs, the public health infrastructure is inadequate for prevention and treatment programmes.

For communicable diseases, the LMICs, especially sub-Saharan African countries, had suboptimal uptake of WHO ART guidelines due to inadequate health systems in those countries. The barriers reported included no operating budget to support scale-up, difficulty transporting samples, delays in commodity procurement and distribution, inadequate laboratory information systems, insufficient trained human resources dedicated for viral load testing, equipment breakdown, delay in equipment repair, inadequate laboratory and storage space to accommodate sample volume, and insufficient viral load testing results management (record keeping and use of results for patient management in healthcare facilities) [34, 65].

For TB prevention, improved access to rapid diagnostics for TB drug resistance and second-line TB treatment was recommended [66]. Based on the 2018 WHO treatment guidelines for multidrug-/rifampicin-resistant tuberculosis (MDR/RR-TB), the capacity for drug susceptibility testing was reportedly insufficient in resource-limited settings, requiring national TB programmes to strengthen their capacity to detect and manage MDR-TB in accordance with the WHO guidelines [67]. Similarly, other studies identified lack of equipment, supplies and human resources as significant barriers to optimal malaria care in Tanzania and Kenya and the PMTCT of HIV in Malawi [68–70].

For MNCH, in the vast majority of countries, ANC is provided free of charge. Accessibility and availability of local transport (for example, visiting a clinic in a distant location or in an unfamiliar part of town), indirect costs associated with transport to and from the clinic in resource-poor settings and the purchase of additional medicines were reported as barriers to ANC engagement in several LMICs [71]. The lack of privacy in the delivery of ANC, rigid and inflexible appointments, lack of medicine and medical equipment at clinics, poor explanation of tests and lack of continuity of care also limit the delivery of quality ANC in LMICs [50, 72, 73]. Studies also suggested the need for a smaller number of evidence-based quality indicators for quality of care in LMICs as opposed to an overwhelming number of indicators in the WHO guidelines [74, 75]. Another study found that a lack of healthcare infrastructure was a significant barrier to the WHO-recommended integrated management of neonatal and childhood illness (IMNCI) strategy compliance in Ethiopia [76].

## Discussion

Public health in LMICs is complex; implementing and taking up broad-sweeping guidelines is even more complex. Our findings reveal that guideline uptake in any one WHO Member State is influenced by a multifactorial interplay of factors such as awareness of guidelines, funding, infrastructure, legislation and regulations. While most of the identified barriers can be directly attributed to challenges within the national health systems context, some barriers are associated with the WHO guidelines themselves.

### Stronger health systems for guideline uptake

Health systems are expected to fulfil three main functions—healthcare delivery, fair treatment to all and meeting health expectations of the population, for which governance is vital. Health system governance is “an aggregation of normative values such as equity and transparency within the political system in which a health system functions” [77]. It involves (1) setting strategic direction and objectives; (2) making policies, laws, rules, regulations or decisions, and raising and deploying resources to accomplish the strategic goals and objectives; and (3) overseeing and making sure that the strategic goals and objectives are accomplished [78]. However, the review findings suggest that governance within the existing health systems in LMICs is weak, as is the guidance provided by WHO guidelines on governance requirements. This includes weak or absent legislation or regulations, poor appreciation of procurement and stock-out challenges, and weak follow-up at the policy and practice levels. This is further exacerbated by a lack of accountability and transparency mechanisms for guideline uptake and implementation within the Member States, particularly LMICs.

Health infrastructure challenges encompassing management and operations issues, systems and technical needs, to community resources were evident in guideline uptake for the health conditions selected in the study. For example, the review findings suggest that lack of infrastructure is a critical barrier for guideline uptake and use in ANC in LMICs [68]. These findings are consistent with the studies undertaken for mobile health (mHealth) intervention implementation in Africa [79]. Studies have found that some of the infrastructural deficits in LMICs may be improved by learning from and building on the successful response to HIV/AIDS through interactions between high-income countries and LMICs [3].

The resource constraints were evident in the evaluation studies undertaken in LMICs, particularly the clinical practice guidelines. For example, with regard to WHO ART guidelines uptake, most of the LMICs did not have a health system in place for guideline uptake and use, requiring domestic, bilateral and multilateral funding to support guideline implementation. In addition, human resources capacity gaps such as poor-quality training, lack of opportunities for skill enhancement and lack of accountability for adherence to guidelines, lack of communication/interprofessional collaboration, and ethnic/cultural differences were cited as barriers to WHO guideline uptake and use in healthcare settings [68]. These challenges reflect the national health systems’ ability to allocate, implement and monitor the guidelines, which historically is beyond WHO’s remit. Nevertheless, the evidence suggests that financial incentives and penalties encourage the uptake of healthy behaviours [80, 81] and compliance with clinical practice guidelines [82] and treatment guidelines [83]. Therefore, WHO guideline developers could potentially explore these possible opportunities for better uptake when developing the guidelines.

Weak health systems hinder the implementation of effective interventions [84]. Poor uptake of guidelines continues to be a significant challenge across health systems, particularly in conflict-hit countries [85]. Evidence suggests that women living in regions with extremely high levels of conflict had decreased odds of meeting the WHO recommendations [86]. For example, study identified several challenges in Kosovo regarding the uptake of maternal health guidelines and their contextualization for local use. The 1998–1999 conflict substantially and adversely affected the healthcare infrastructure in Kosovo, which has resulted in an inability to monitor the quality of care across the country. Furthermore, the impact on infrastructure has affected the ability for consistent access to required medications and smooth transfer of patients from rural to urban centres [87]. This demonstrates the role of stable and robust health systems as a critical determinant for guideline uptake in LMICs.

### Addressing WHO guideline complexities, weaknesses and implementation guidance

Notwithstanding health systems challenges, the review findings suggest that the WHO guidelines themselves were either unclear or weak and were technically challenging. Evidence from implementation research has shown that detailed implementation plans are often necessary for local policy-makers to use WHO guidelines. Studies have also found that end-users’ adherence to and uptake of guidelines are negatively affected by guidelines without adequate implementation plans [10, 88, 89].

Further, implementation advice was often not provided, especially in terms of sustained capacity-building, which limited stakeholder engagement [90], and guidelines were often too technical and did not cater to end-user needs. The review found these limitations, for example, in PPH guidelines [34] and maternal and newborn care guidelines in health facilities [74], and this was consistent with studies that reported WHO’s confusing guidance on masks in the COVID-19 pandemic [91]. Similar to many empirical research studies on a variety of health conditions in this review, the WHO evaluation office commissioned an evaluation of the impact of the WHO publications, which also found that WHO products were often described as “too long, too technical” and needed to be tailored to different audiences [92].

Our findings also suggest that WHO guidelines were being used as a reference by Member States when developing their national guidelines. However, guideline dissemination and the monitoring and evaluation of guideline uptake were not well documented by WHO or the Member States for optimizing guideline uptake. A WHO-commissioned assessment of the contribution of WHO guidelines to improving reproductive, maternal and newborn health in the South-East Asia Region found that WHO engaged its intended audience by deploying various dissemination means (e.g. electronic, regional meetings). However, the process was not well monitored or documented regarding the distribution of emails, downloads from websites and distribution of printed copies [93]. Similarly, another review found a lack of well-documented adaptation methodologies in national HIV and/or TB guidelines and the need for a standardized and systematic framework for guideline adaptation and improved reporting of processes for guideline use [94]. Further, the WHO guidelines often do not include feedback mechanisms for compliance between WHO and Member States, significantly restricting the ability to understand, monitor and evaluate guideline uptake.

### Policy implications

The WHO GPW13 focuses on Triple Billion targets to achieve measurable impacts on population health at the country level. The Triple Billion targets include one billion more people benefiting from universal health coverage, one billion more people better protected from health emergencies, and one billion more people enjoying better health and well-being. WHO’s guidance is vital in achieving the Triple Billion targets and measurable impacts on population health for the Member States. As the review findings determine the direct correlation between guideline uptake and health systems, the organization, while producing evidence-based guidelines for better health outcomes in Member States, should continue to encourage the building of stronger health systems to optimize the WHO guidelines in the Member States. Additionally, WHO should monitor and evaluate the uptake of its guidelines with either existing or new monitoring, evaluation and learning frameworks and feedback loops between WHO and Member States for optimizing WHO guideline uptake in Member States. WHO SMART (standards-based, machine-readable, adaptive, requirements-based and testable) guidelines are a comprehensive set of reusable digital health components as a way forward for optimizing guideline uptake [95].

### Future research recommendations

Guideline developers should work collaboratively with guideline implementors and researchers to design and conduct evaluations of guideline implementation, especially in the LMICs, to identify additional contextually sensitive barriers and facilitators. Targeted implementation strategies could then be developed and tested in the local settings. Also, the funding organizations should focus on and encourage these evaluation and monitoring studies. As for addressing the barriers related to the WHO guidelines, researchers should focus on the impact of different formats and reporting characteristics of the guideline recommendations, and engage with guideline implementors and developers to identify the optimal formats that they could accept.

### Limitations of the study

WHO maintains the Global Index Medicus (GIM) data, which provides worldwide access to biomedical and public health literature produced by and within LMICs. By not including GIM in our search strategy, the review may have missed some critical articles from the LMICs. Also, our screening criteria included literature in English only, restricting the review to articles published in the English language.

## Conclusions

The challenges for WHO guideline uptake reflect the health systems’ ability to allocate, implement and monitor the guidelines. Historically this is beyond the remit of WHO, but Member States could benefit from WHO implementation guidance on requirements and needs for successful deployment of WHO’s NSPs, including the guidelines. The impact on health outcomes is derived primarily from guideline implementation; however, the extent to which guidelines are implemented in countries, and the quality of that implementation, largely remains unknown. WHO guidelines are referenced and adapted to a large extent at the country level into national policies, strategies, plans and clinical guidelines.

## Supplementary Information

**Additional file 1.** Search strategy.

## Data Availability

Not applicable.

## References

[1] WHO. Supporting national health policies, strategies, plans 2021 [Available from: https://www.who.int/activities/supporting-national-health-policies-strategies-plans.

[2] WHO. Evaluation of WHO’s Normative Function. 2017.

[3] Olayemi E, Asare EV, Benneh-Akwasi Kuma AA (2017). Guidelines in lower-middle income countries. Br J Haematol.

[4] Dans AL, Dans LF, Lanas F (2020). Guidelines in low and middle income countries paper 1: scoping clinical practice guidelines in Chile and the Philippines. J Clin Epidemiol.

[5] Gunderman DV, Eric. Low- and middle-income countries lack access to big data analysis—here’s how to fill the gap2021. Available from: https://theconversation.com/low-and-middle-income-countries-lack-access-to-big-data-analysis-heres-how-to-fill-the-gap-159412.

[6] Haycox A (2018). Should low- and middle-income countries adopt clinical guidelines developed in ‘rich’ countries?. Pharmacoeconomics.

[7] WHO. Norms and standards: biotherapeutics and biosimilars. WHO Drug Information. 2015;29(2):138–41.

[8] WHO. Thirteenth general programme of work, 2019–2023: promote health, keep the world safe, serve the vulnerable. World Health Organization; 2019.

[9] Oxman AD, Fretheim A, Schünemann HJ (2006). Improving the use of research evidence in guideline development: introduction. Health Res Policy Syst.

[10] Wang Z, Grundy Q, Parker L, Bero L (2020). Variations in processes for guideline adaptation: a qualitative study of World Health Organization staff experiences in implementing guidelines. BMC Public Health.

[11] Gagliardi AR, Brouwers MC, Palda VA, Lemieux-Charles L, Grimshaw JM (2011). How can we improve guideline use? A conceptual framework of implementability. Implement Sci.

[12] WHO. Monitoring, Evaluation, and Learning White Paper. 2021.

[13] Sinclair D, Isba R, Kredo T, Zani B, Smith H, Garner P (2013). World Health Organization guideline development: an evaluation. PLoS ONE.

[14] Lockwood C, Dos Santos KB, Pap R (2019). Practical guidance for knowledge synthesis: scoping review methods. Asian Nurs Res.

[15] Daudt HL, van Mossel C, Scott SJ. Enhancing the scoping study methodology: a large, interprofessional team’s experience with Arksey and O’Malley’s framework. BMC Med Res Methodol. 2013;13. 10.1186/1471-2288-13-48 PMC3614526 23522333

[16] Wang X, Zhou Q, Chen Y, Yang N, Pottie K, Xiao Y (2020). Using RIGHT (Reporting Items for Practice Guidelines in Healthcare) to evaluate the reporting quality of WHO guidelines. Health Res Policy Syst.

[17] Tricco AC, Lillie E, Zarin W, O'Brien KK, Colquhoun H, Levac D (2018). PRISMA extension for scoping reviews (PRISMA-ScR): checklist and explanation. Ann Intern Med.

[18] Munn Z, Peters MD, Stern C, Tufanaru C, McArthur A, Aromataris E (2018). Systematic review or scoping review? Guidance for authors when choosing between a systematic or scoping review approach. BMC Med Res Methodol.

[19] Page MJ, McKenzie JE, Bossuyt PM, Boutron I, Hoffmann TC, Mulrow CD (2021). Updating guidance for reporting systematic reviews: development of the PRISMA 2020 statement. J Clin Epidemiol.

[20] Reporting guidelines under development: Preferred Reporting Items for Systematic Reviews and Meta-Analysis extension for Scoping Reviews (PRISMA-ScR): The EQUATOR Network; 2017. Available from: http://www.equator-network.org/library/reporting-guidelines-under-development/#55.

[21] Faulkner SL, Trotter SP. Theoretical saturation. The International encyclopedia of communication research methods. 2017:1–2.

[22] Arksey H, O'Malley L (2005). Scoping studies: towards a methodological framework. Int J Soc Res Methodol.

[23] Gostin LO (2000). Public health law in a new century: part I: law as a tool to advance the community’s health. JAMA.

[24] Beier JC, Keating J, Githure JI, Macdonald MB, Impoinvil DE, Novak RJ (2008). Integrated vector management for malaria control. Malar J.

[25] WHO. Assessing national capacity for the prevention and control of noncommunicable diseases: report of the 2019 global survey. 2020.

[26] Nyaaba GN, Stronks K, Aikins A-G, Kengne AP, Agyemang C (2017). Tracing Africa’s progress towards implementing the Non-Communicable Diseases Global action plan 2013–2020: a synthesis of WHO country profile reports. BMC Public Health.

[27] Kaltenbrun TA, du Plessis LM, Drimie S (2020). A qualitative analysis of perceptions of various stakeholders on nutrition-sensitive agricultural interventions, including the taxation on sugar-sweetened beverages (SSBs), to improve overall health and nutrition in South Africa. BMC Public Health.

[28] Pati MK, Swaroop N, Kar A, Aggarwal P, Jayanna K, Van Damme W (2020). A narrative review of gaps in the provision of integrated care for noncommunicable diseases in India. Public Health Rev.

[29] Hossain M, Chisti MJ, Hossain MI, Mahfuz M, Islam MM, Ahmed T (2017). Efficacy of World Health Organization guideline in facility-based reduction of mortality in severely malnourished children from low and middle income countries: a systematic review and meta-analysis. J Paediatr Child Health.

[30] Mejia LA, Kuo WY, Beltran-Velazquez F (2019). Provision of micronutrients in coexisting public health programs and risk of excessive intake: regulatory considerations. Ann N Y Acad Sci.

[31] Organization WH. WHO recommendations: optimizing health worker roles to improve access to key maternal and newborn health interventions through task shifting: World Health Organization; 2012. 23844452

[32] Colvin CJ, de Heer J, Winterton L, Mellenkamp M, Glenton C, Noyes J (2013). A systematic review of qualitative evidence on barriers and facilitators to the implementation of task-shifting in midwifery services. Midwifery.

[33] Shilton S, Chandra-Mouli V, Paul S, Denno DM. Facilitators and barriers in the utilization of World Health Organization’s Preventing Early Pregnancy Guidelines in formulating laws, policies and strategies: what do stakeholders in Ethiopia say? Int J Adolesc Med Health. 2019;1(ahead-of-print). 10.1515/ijamh-2019-0028 31271553

[34] Vogel JP, Moore JE, Timmings C, Khan S, Khan DN, Defar A (2016). Barriers, facilitators and priorities for implementation of WHO maternal and perinatal health guidelines in four lower-income countries: a GREAT network research activity. PLoS ONE.

[35] Khosla R, Banerjee J, Chou D, Say L, Fried ST (2017). Gender equality and human rights approaches to female genital mutilation: a review of international human rights norms and standards. Reprod Health.

[36] Smith B, Kwok C, Nutbeam D (2011). WHO Health Promotion Glossary: new terms. Health Promotion Int.

[37] Smith BJ, Tang KC, Nutbeam D (2006). WHO health promotion glossary: new terms. Health Promot Int.

[38] Nasser SMU, Cooke G, Kranzer K, Norris SL, Olliaro P, Ford N (2015). Strength of recommendations in WHO guidelines using GRADE was associated with uptake in national policy. J Clin Epidemiol.

[39] Church K, Kiweewa F, Dasgupta A, Mwangome M, Mpandaguta E, Gómez-Olivé FX (2015). A comparative analysis of national HIV policies in six African countries with generalized epidemics. Bull World Health Organ.

[40] Finocchario-Kessler S, Clark KF, Khamadi S, Gautney BJ, Okoth V, Goggin K (2016). Progress toward eliminating mother to child transmission of HIV in Kenya: review of treatment guideline uptake and pediatric transmission at four government hospitals between 2010 and 2012. AIDS Behav.

[41] Tudor Car L, Brusamento S, Elmoniry H, van Velthoven MH, Pape UJ, Welch V (2013). The uptake of integrated perinatal prevention of mother-to-child HIV transmission programs in low-and middle-income countries: a systematic review. PLoS ONE.

[42] Doherty T, Chopra M, Jackson D, Goga A, Colvin M, Persson L-A (2007). Effectiveness of the WHO/UNICEF guidelines on infant feeding for HIV-positive women: results from a prospective cohort study in South Africa. AIDS.

[43] Chinkonde JR, Sundby J, de Paoli M, Thorsen VC (2010). The difficulty with responding to policy changes for HIV and infant feeding in Malawi. Int Breastfeed J.

[44] Ngoma MS, Misir A, Mutale W, Rampakakis E, Sampalis JS, Elong A (2015). Efficacy of WHO recommendation for continued breastfeeding and maternal cART for prevention of perinatal and postnatal HIV transmission in Zambia. J Int AIDS Soc.

[45] Xue P, Ng MTA, Qiao Y (2020). The challenges of colposcopy for cervical cancer screening in LMICs and solutions by artificial intelligence. BMC Med.

[46] Samnani AABA, Rizvi N, Ali TS, Abrejo F (2017). Barriers or gaps in implementation of misoprostol use for post-abortion care and post-partum hemorrhage prevention in developing countries: a systematic review. Reprod Health.

[47] Chu CS, Brhlikova P, Pollock AM (2012). Rethinking WHO guidance: review of evidence for misoprostol use in the prevention of postpartum haemorrhage. J R Soc Med.

[48] Moore JE, Uka S, Vogel JP, Timmings C, Rashid S, Gülmezoglu AM (2016). Navigating barriers: two-year follow up on recommendations to improve the use of maternal health guidelines in Kosovo. BMC Public Health.

[49] Braddick L, Tuckey V, Abbas Z, Lissauer D, Ismail K, Manaseki-Holland S (2016). A mixed-methods study of barriers and facilitators to the implementation of postpartum hemorrhage guidelines in Uganda. Int J Gynecol Obstet.

[50] Downe S, Finlayson K, Tunçalp Ö, Gülmezoglu AM. Provision and uptake of routine antenatal services: a qualitative evidence synthesis. Cochrane Database Syst Rev. 2019(6). 10.1002/14651858.CD012392.pub2 PMC6564082 31194903

[51] Kumar S, Yadav V, Balasubramaniam S, Jain Y, Joshi CS, Saran K (2016). Effectiveness of the WHO SCC on improving adherence to essential practices during childbirth, in resource constrained settings. BMC Pregnancy Childbirth.

[52] Ansah Manu A, ten Asbroek A, Soremekun S, Gyan T, Weobong B, Tawiah-Agyemang C (2014). Evaluating the implementation of community volunteer assessment and referral of sick babies: lessons learned from the Ghana Newhints home visits cluster randomized controlled trial. Health Policy Plan.

[53] Govere SM, Chimbari MJ (2020). The evolution and adoption of World Health Organization policy guidelines on antiretroviral therapy initiation in sub-Saharan Africa: a scoping review. South Afr J HIV Med.

[54] WHO. The European Action Plan for Strengthening Public Health Capacities and Services. Copenhagen, Denmark: WHO Regional Office for Europe; 2012.

[55] Chanda E, Mzilahowa T, Chipwanya J, Mulenga S, Ali D, Troell P (2015). Preventing malaria transmission by indoor residual spraying in Malawi: grappling with the challenge of uncertain sustainability. Malar J.

[56] Stover J, Gopalappa C, Mahy M, Doherty MC, Easterbrook PJ, Weiler G (2014). The impact and cost of the 2013 WHO recommendations on eligibility for antiretroviral therapy. AIDS.

[57] Stanecki K, Daher J, Stover J, Beusenberg M, Souteyrand Y, Calleja JMG (2010). Antiretroviral therapy needs: the effect of changing global guidelines. Sex Transmitted Infect..

[58] Hodges-Mameletzis I, Dalal S, Msimanga-Radebe B, Rodolph M, Baggaley R (2018). Going global: the adoption of the World Health Organization’s enabling recommendation on oral pre-exposure prophylaxis for HIV. Sex Health.

[59] Singhroy DN, MacLean E, Kohli M, Lessem E, Branigan D, England K (2020). Adoption and uptake of the lateral flow urine LAM test in countries with high tuberculosis and HIV/AIDS burden: current landscape and barriers. Gates Open Res..

[60] Downs SM, Singh A, Gupta V, Lock K, Ghosh-Jerath S (2015). The need for multisectoral food chain approaches to reduce trans fat consumption in India. BMC Public Health.

[61] Dzudie A, Njume E, Abanda M, Aminde L, Hamadou B, Dzekem B (2020). Availability, cost and affordability of essential cardiovascular disease medicines in the south west region of Cameroon: preliminary findings from the Cameroon science for disease study. PLoS ONE.

[62] Nsabagasani X, Hansen E, Mbonye A, Ssengooba F, Muyinda H, Mugisha J (2015). Explaining the slow transition of child-appropriate dosage formulations from the global to national level in the context of Uganda: a qualitative study. J Pharm Policy Pract.

[63] Kraft JM, Oduyebo T, Jatlaoui TC, Curtis KM, Whiteman MK, Zapata LB (2018). Dissemination and use of WHO family planning guidance and tools: a qualitative assessment. Health Res Policy Syst.

[64] Turnock BJ (2001). Public health: what it is and how it works.

[65] Lecher S, Ellenberger D, Kim AA, Fonjungo PN, Agolory S, Borget MY (2015). Scale-up of HIV viral load monitoring—seven sub-Saharan African countries. Morb Mortal Wkly Rep.

[66] Jones-López EC, Ayakaka I, Levin J, Reilly N, Mumbowa F, Dryden-Peterson S (2011). Effectiveness of the standard WHO recommended retreatment regimen (category II) for tuberculosis in Kampala, Uganda: a prospective cohort study. PLoS Med.

[67] Van Deun A, Decroo T, Tahseen S, Trébucq A, Schwoebel V, Ortuno-Gutierrez N, et al. World Health Organization 2018 treatment guidelines for rifampicin-resistant tuberculosis: uncertainty, potential risks and the way forward. Elsevier; 2020. 10.1016/j.ijantimicag.2019.10.003 31626907

[68] Ritchie LMP, Khan S, Moore JE, Timmings C, van Lettow M, Vogel JP (2016). Low-and middle-income countries face many common barriers to implementation of maternal health evidence products. J Clin Epidemiol.

[69] Nadjm B, Amos B, Mtove G, Ostermann J, Chonya S, Wangai H (2010). WHO guidelines for antimicrobial treatment in children admitted to hospital in an area of intense *Plasmodium falciparum* transmission: prospective study. BMJ.

[70] Tlhajoane M, Masoka T, Mpandaguta E, Rhead R, Church K, Wringe A (2018). A longitudinal review of national HIV policy and progress made in health facility implementation in Eastern Zimbabwe. Health Res Policy Syst.

[71] Doku DT, Neupane S (2017). Survival analysis of the association between antenatal care attendance and neonatal mortality in 57 low-and middle-income countries. Int J Epidemiol.

[72] Mchenga M, Burger R, Von Fintel D (2019). Examining the impact of WHO’s Focused Antenatal Care policy on early access, underutilisation and quality of antenatal care services in Malawi: a retrospective study. BMC Health Serv Res.

[73] Roberts J, Hopp Marshak H, Sealy DA, Manda-Taylor L, Mataya R, Gleason P (2017). The role of cultural beliefs in accessing antenatal care in Malawi: a qualitative study. Public Health Nurs.

[74] Chang KT, Hossain P, Sarker M, Montagu D, Chakraborty NM, Sprockett A (2020). Translating international guidelines for use in routine maternal and neonatal healthcare quality measurement. Glob Health Action.

[75] Zhang S, Incardona B, Qazi SA, Stenberg K, Campbell H, Nair H, et al. Cost–effectiveness analysis of revised WHO guidelines for management of childhood pneumonia in 74 Countdown countries. J Global Health. 2017;7(1). 10.7189/jogh.07.010409 PMC5344007 28400955

[76] Abebe AM, Kassaw MW, Mengistu FA (2019). Assessment of factors affecting the implementation of integrated management of neonatal and childhood illness for treatment of under five children by health professional in health care facilities in Yifat Cluster in North Shewa Zone, Amhara Region, Ethiopia. Int J Pediatr.

[77] Balabanova D, Mills A, Conteh L, Akkazieva B, Banteyerga H, Dash U (2013). Good Health at Low Cost 25 years on: lessons for the future of health systems strengthening. The Lancet.

[78] Shukla M, Colindres H, Orton M. How to govern the health sector and its institutions effectively. The E_Manager, Management Strategies for Improving Health Services. 2013.

[79] Aranda-Jan CB, Mohutsiwa-Dibe N, Loukanova S (2014). Systematic review on what works, what does not work and why of implementation of mobile health (mHealth) projects in Africa. BMC Public Health.

[80] Adams J, Giles EL, Robalino S, McColl E, Sniehotta FF (2012). A systematic review of the use of financial incentives and penalties to encourage uptake of healthy behaviors: protocol. Syst Rev.

[81] Giles EL, Sniehotta FF, McColl E, Adams J (2015). Acceptability of financial incentives and penalties for encouraging uptake of healthy behaviours: focus groups. BMC Public Health.

[82] Bai J, Bundorf K, Bai F, Tang H, Xue D (2019). Relationship between physician financial incentives and clinical pathway compliance: a cross-sectional study of 18 public hospitals in China. BMJ Open.

[83] Komasi S, Saeidi M, Sariaslani P, Soroush A (2018). Applying behavioural incentives to increase adherence to maintenance treatment. Malays J Med Sci MJMS.

[84] Bosch-Capblanch X, Lavis JN, Lewin S, Atun R, Røttingen J-A, Dröschel D (2012). Guidance for evidence-informed policies about health systems: rationale for and challenges of guidance development. PLoS Med.

[85] Gupta S, Rai N, Bhattacharrya O, Cheng AY, Connelly KA, Boulet L-P (2016). Optimizing the language and format of guidelines to improve guideline uptake. CMAJ.

[86] Ziegler BR, Kansanga M, Sano Y, Kangmennaang J, Kpienbaareh D, Luginaah I (2020). Antenatal care utilization in the fragile and conflict-affected context of the Democratic Republic of the Congo. Soc Sci Med.

[87] Straus SE, Moore JE, Uka S, Marquez C, Gülmezoglu AM (2013). Determinants of implementation of maternal health guidelines in Kosovo: mixed methods study. Implement Sci.

[88] Li MY, Kelly J, Subhi R, Were W, Duke T (2013). Global use of the WHO pocket book of hospital care for children. Paediatr Int Child Health.

[89] Gray AZ, Soukaloun D, Soumphonphakdy B, Duke T (2015). Implementing WHO hospital guidelines improves quality of paediatric care in central hospitals in Lao PDR. Tropical Med Int Health.

[90] Burda B, Chambers A, Johnson J (2014). Appraisal of guidelines developed by the World Health Organization. Public Health.

[91] Chan A, Leung C, Lam T, Cheng K. To wear or not to wear: WHO’s confusing guidance on masks in the COVID-19 pandemic. BMJ opinion. 2020.

[92] WHO. Evaluation of the Impact of WHO Publications. 2016.

[93] WHO. Evaluation of the adaptation and use of WHO guidelines on Reproductive, Maternal and Newborn Health (RMNH) in the WHO South-East Asia (SEA). Geneva World Health Organisation; 2020.

[94] Godah MW, Khalek RAA, Kilzar L, Zeid H, Nahlawi A, Lopes LC (2016). A very low number of national adaptations of the World Health Organization guidelines for HIV and tuberculosis reported their processes. J Clin Epidemiol.

[95] Mehl G, Tunçalp Ö, Ratanaprayul N, Tamrat T, Barreix M, Lowrance D (2021). WHO SMART guidelines: optimising country-level use of guideline recommendations in the digital age. The Lancet Digital Health.

