# Supplementary material for: Improving WHO’s understanding of WHO guideline uptake and use in Member States: a scoping review
Source: Health Res Policy Syst. 2022 Sep 7;20:98. doi: 10.1186/s12961-022-00899-y (PMC9449928; doi:10.1186/s12961-022-00899-y)
Supplement: Supplementary file 1 — Additional file 1. Search strategy. [file 12961_2022_899_MOESM1_ESM.docx]

**Supplementary File 1: Search Strategy**

1. **CINAHL search history**

| **Query/Details** |
| --- |
| 1. (MH "World Health Organization+") 2. TX "World Health Organization" 3. TX "the WHO" 4. **#1 OR #2 OR #3** 5. (MH "Practice Guidelines") 6. TI guideline* OR AB guideline* 7. TI recommendation* OR AB recommendation* 8. TI guidance OR AB guidance 9. TI manual* OR AB manual* 10. TI "rapid advice" OR AB "rapid advice" 11. TI statement* OR AB statement* 12. **#5 OR #6 OR #7 OR #8 OR #9 OR #10 OR #11** 13. (MH "Guideline Adherence") 14. (MH "Implementation Science") 15. (MH "Program Implementation") 16. TI adopt* OR AB adopt* 17. TI adapt* OR AB adapt* 18. TI contextuali* OR AB contextuali* 19. TI implement* OR AB implement* 20. TI utili* OR AB utili* 21. TI uptake OR AB uptake 22. TI adhere* OR AB adhere* 23. TI conform* OR AB conform* 24. TI complian* OR AB complian* 25. TI comply OR AB comply 26. TI concord* OR AB concord* 27. TI accept* OR AB accept* 28. TI audit* OR AB audit* 29. TI monitor* OR AB monitor* 30. TI evaluat* OR AB evaluat* 31. TI assess* OR AB assess* 32. **#13 OR #14 OR #15 OR #16 OR #17 OR #18 OR #19 OR #20 OR #21 OR #22 OR #23 OR #24 OR #25 OR #26 OR #27 OR #28 OR #29 OR #30 OR #31** 33. (MH "Developing Countries") 34. TI "developing country" OR AB "developing country" 35. TI "developing countries" OR AB "developing countries" 36. TI "low-income country" OR AB "low-income country" 37. TI "low-income countries" OR AB "low-income countries" 38. TI "middle-income country" OR AB "middle-income country" 39. TI "middle-income countries" OR AB "middle-income countries" 40. TI ( "low- and middle-income country" ) OR AB ( "low- and middle-income country") 41. ( "low- and middle-income countries" ) OR AB ( "low- and middle-income countries" ) 42. TI "LMIC" OR AB "LMIC" 43. **#33 OR #34 OR #35 OR #36 OR #37 OR #38 OR #39 OR #40 OR #41 OR #42** 44. **#4 AND #12 AND #32 AND #43** |
| We used (MH "Program Implementation") in CINAHL since it did not have the term "Health Plan Implementation" as a Mesh term. Hyphen or no hyphen gives same result in CINAHL (TI "low income country" OR AB "low income country"=TI "low-income country" OR AB "low-income country"). |

1. **Cochrane Library (CENTRAL) search history**

| **Query/Details** |
| --- |
| 1. MeSH descriptor: [World Health Organization] explode all trees 2. ("World Health Organization") 3. ("the WHO") 4. **#1 OR #2 OR #3** 5. MeSH descriptor: [Guidelines as Topic] explode all trees 6. (guideline*):ti,ab,kw 7. (recommendation*):ti,ab,kw 8. (guidance):ti,ab,kw 9. (manual*):ti,ab,kw 10. ("rapid advice"):ti,ab,kw 11. (statement*):ti,ab,kw 12. **#5 OR #6 OR #7 OR #8 OR #9 OR #10 OR #11** 13. MeSH descriptor: [Guideline Adherence] explode all trees 14. MeSH descriptor: [Implementation Science] explode all trees 15. MeSH descriptor: [Health Plan Implementation] explode all trees 16. (adopt*):ti,ab,kw 17. (adapt*):ti,ab,kw 18. (contextuali*):ti,ab,kw 19. (implement*):ti,ab,kw 20. (utili*):ti,ab,kw 21. (uptake):ti,ab,kw 22. (adhere*):ti,ab,kw 23. (conform*):ti,ab,kw 24. (complian*):ti,ab,kw 25. (comply):ti,ab,kw 26. (concord*):ti,ab,kw 27. (concord*):ti,ab,kw 28. (audit*):ti,ab,kw 29. (monitor*):ti,ab,kw 30. (evaluat*):ti,ab,kw 31. (assess*):ti,ab,kw 32. **#13 OR #14 OR #15 OR #16 OR #17 OR #18 OR #19 OR #20 OR #21 OR #22 OR #23 OR #24 OR #25 OR #26 OR #27 OR #28 OR #29 OR #30 OR #31** 33. MeSH descriptor: [Developing Countries] explode all trees 34. ("developing country"):ti,ab,kw 35. ("developing countries"):ti,ab,kw 36. ("low-income country"):ti,ab,kw 37. ("low-income countries"):ti,ab,kw 38. ("middle-income country"):ti,ab,kw 39. ("middle-income countries"):ti,ab,kw 40. ("low- and middle-income country"):ti,ab,kw 41. ("low- and middle-income countries"):ti,ab,kw 42. ("LMIC"):ti,ab,kw 43. **#33 OR #34 OR #35 OR #36 OR #37 OR #38 OR #39 OR #40 OR #41 OR #42** 44. **#4 AND #14 AND #32 AND #43** |
| * Hyphen or no hyphen gives same result in Cochrane Library. |

1. **OVID – MEDLINE**

| **Query/Details** |
| --- |
| 1. exp World Health Organization/ 2. World Health Organization.mp. 3. the WHO.mp. 4. **#1 OR #2 OR #3** 5. exp Guideline/ 6. guideline*.ti,ab. 7. recommendation*.ti,ab. 8. guidance.ti,ab. 9. manual*.ti,ab. 10. rapid advice.ti,ab. 11. statement*.ti,ab. 12. **#5 OR #6 OR #7 OR #8 OR #9 OR #10 OR #11** 13. exp Guideline Adherence/ 14. exp Health Plan Implementation/ 15. exp Implementation Science/ 16. adopt*.ti,ab. 17. adapt*.ti,ab. 18. contextuali*.ti,ab. 19. implement*.ti,ab. 20. utili*.ti,ab. 21. uptake.ti,ab. 22. adhere*.ti,ab. 23. conform*.ti,ab. 24. complian*.ti,ab. 25. comply.ti,ab. 26. concord*.ti,ab. 27. accept*.ti,ab. 28. audit*.ti,ab. 29. monitor*.ti,ab. 30. evaluat*,ti.ab. 31. assess*.ti,ab. 32. **#13 OR #14 OR #15 OR #16 OR #17 OR #18 OR #19 OR #20 OR #21 OR #22 OR #23 OR #24 OR #25 OR #26 OR #27 OR #28 OR #29 OR #30 OR #31** 33. exp Developing Countries/ 34. developing countr*.ti,ab. 35. low-income countr*.ti,ab. 36. middle-income countr*.ti,ab. 37. (low and middle income countr*).ti,ab. 38. LMIC.ti,ab. 39. **#33 OR #34 OR #35 OR #36 OR #37 OR #38** 40. **#4 AND #12 AND #32 AND #39** |
| Hyphen or no hyphen gives same result. |

1. **OVID – EMBASE**

| **Query/Details** |
| --- |
| 1. exp World Health Organization/ 2. World Health Organization.mp. 3. the WHO.mp. 4. **#1 OR #2 OR #3** 5. exp practice guideline/ 6. guideline*.ti,ab. 7. recommendation*.ti,ab. 8. guidance.ti,ab. 9. manual*.ti,ab. 10. rapid advice.ti,ab. 11. statement*.ti,ab. 12. **#5 OR #6 OR #7 OR #8 OR #9 OR #10 OR #11** 13. exp protocol compliance/ 14. exp implementation science/ 15. adopt*.ti,ab. 16. adapt*.ti,ab. 17. contextuali*.ti,ab. 18. implement*.ti,ab. 19. utili*.ti,ab. 20. uptake.ti,ab. 21. adhere*.ti,ab. 22. conform*.ti,ab. 23. complian*.ti,ab. 24. comply.ti,ab. 25. concord*.ti,ab. 26. accept*.ti,ab. 27. audit*.ti,ab. 28. monitor*.ti,ab. 29. evaluat*.ti,ab. 30. assess*.ti,ab. 31. **#13 OR #14 OR #15 OR #16 OR #17 OR #18 OR #19 OR #20 OR #21 OR #22 OR #23 OR #24 OR #25 OR #26 OR #27 OR #28 OR #29 OR #30** 32. exp developing country/ 33. Developing Countr*.ti,ab. 34. low-income countr*.ti,ab. 35. middle-income countr*.ti,ab. 36. (low and middle income countr*).ti,ab. 37. LMIC.ti,ab. 38. **#32 OR #33 OR #34 OR #35 OR #36 OR #37** 39. **#4 AND #12 AND #31 AND #38** |
| Hyphen or no hyphen gives same result. |

1. **SCOPUS**

| **Query/Details** |
| --- |
| 1. TITLE-ABS-KEY: (world AND health AND organization) 2. TITLE-ABS-KEY: WHO 3. TITLE-ABS-KEY: the WHO 4. **#1 OR #2 OR #3** 5. TITLE-ABS-KEY: guideline* 6. TITLE-ABS-KEY: recommendation* 7. TITLE-ABS-KEY: guidance 8. TITLE-ABS-KEY: manual* 9. TITLE-ABS-KEY: (rapid AND advice) 10. TITLE-ABS-KEY: statement* 11. **#5 OR #6 OR #7 OR #8 OR #9 OR #10** 12. TITLE-ABS-KEY: (guideline AND adherence) 13. TITLE-ABS-KEY: adopt* 14. TITLE-ABS-KEY: adapt* 15. TITLE-ABS-KEY: contextuali* 16. TITLE-ABS-KEY: implement* 17. TITLE-ABS-KEY: utili* 18. TITLE-ABS-KEY: uptake 19. TITLE-ABS-KEY: adhere* 20. TITLE-ABS-KEY: conform* 21. TITLE-ABS-KEY: comply 22. TITLE-ABS-KEY: concord 23. TITLE-ABS-KEY: accept* 24. TITLE-ABS-KEY: audit* 25. TITLE-ABS-KEY: monitor* 26. TITLE-ABS-KEY: evaluat* 27. TITLE-ABS-KEY: assess* 28. **#12 OR #13 OR #14 OR #15 OR #16 OR #17 OR #18 OR #19 OR #20 OR #21 OR #22 OR #23 OR #24 OR #25 OR #26 OR #27** 29. TITLE-ABS-KEY: (developing AND countr*) 30. TITLE-ABS-KEY: (low-income AND countr*) 31. TITLE-ABS-KEY: (middle-income AND countr*) 32. TITLE-ABS-KEY: (low AND middle AND income AND countr*) 33. TITLE-ABS-KEY: lmic 34. **#29 OR #30 OR #31 OR #32 OR #33** 35. **#4 AND #11 AND #28 AND #34** |
